# Aerogels Based on Chitosan and Collagen Modified with Fe_2_O_3_ and Fe_3_O_4_ Nanoparticles: Fabrication and Characterization

**DOI:** 10.3390/polym17020133

**Published:** 2025-01-08

**Authors:** Carmen Mª Granados-Carrera, Daniel Castro-Criado, Johar Amin Ahmed Abdullah, Mercedes Jiménez-Rosado, Víctor M. Perez-Puyana

**Affiliations:** 1 Department of Chemical Engineering, Faculty of Chemistry, University of Seville, 41012 Seville, Spain; cargracar@alum.us.es (C.M.G.-C.); dancascri@alum.us.es (D.C.-C.); jabdullah@us.es (J.A.A.A.); 2 Department of Applied Chemistry and Physics, Faculty of Biological and Ambiental Sciences, University of León, 24009 León, Spain; 3 Department of Engineering and Materials Science and Transportation, University of Seville, 41092 Seville, Spain

**Keywords:** chitosan, collagen, aerogels, tissue engineering, nanoparticles, iron oxide, biomaterials

## Abstract

The necessity to mitigate the intrinsic issues associated with tissue or organ transplants, in order to address the rising prevalence of diseases attributable to increased life expectancy, provides a rationale for the pursuit of innovation in the field of biomaterials. Specifically, biopolymeric aerogels represent a significant advancement in the field of tissue engineering, offering a promising solution for the formation of temporary porous matrices that can replace damaged tissues. However, the functional characteristics of these materials are inadequate, necessitating the implementation of matrix reinforcement methods to enhance their performance. In this study, chemical and green iron oxide nanoparticles, previously synthesized and documented in existing research, were incorporated into hybrid aerogels combining collagen (C) and chitosan (CH). The characterization of these aerogels was conducted through rheological, microstructural, and functional analyses. The results demonstrate that the incorporation of iron oxide nanoparticles has a significant influence on the properties of the aerogels fabricated with them. In particular, the incorporation of these nanoparticles has been observed to modify the mechanical properties, with an increase in strength and porosity that may support cell proliferation.

## 1. Introduction

The exponential growth in the number of individuals with one or more chronic diseases as a consequence of the increase in global life expectancy is resulting in a corresponding rise in the number of organ transplants required. However, the demand for these transplants cannot be met due to the insufficient quantity of available organs [1,2]. Consequently, scientists are concentrating their efforts on the creation of innovative biomaterials that can regenerate, replace, and repair tissues, with the aim of reducing the risk of infections associated with conventional treatments [3,4]. In this context, biomaterials are a diverse range of materials, including metal, ceramic, and polymeric compounds, which are designed to be biocompatible (to avoid adverse reactions when in contact with host tissue), bioactive (to stimulate tissue responses), biodegradable (to disappear after the tissue is healed), and sterilizable, allowing their use in tissue engineering for the replacement of damaged structures [5,6,7,8]. In particular, the main objective of tissue engineering is the development of scaffolds that mimic the dynamics of the extracellular matrix (ECM) and regulate cell adhesion, migration, and other processes [9,10]. However, this is not as simple as it seems since there are tissues that require specific requirements after transplantation (such as heart valves or blood vessels, for example); therefore, knowledge of the mechanical–functional properties of these tissues will be necessary [11,12,13].

Biomaterials can be synthesized by a number of different fabrication methods, including conventional techniques (e.g., casting, phase separation and lyophilization, synthetization, auto-assembly, or electrospinning) or additive techniques (e.g., stereolithography, fused-deposition modeling (FDM), or three-dimensional printing) [8,14,15]. However, among the different biomaterials, aerogels stand out due to their distinctive physical structure, comprising three-dimensional porous solid networks with an exceptionally high specific surface area [16]. Specifically, this type of material can be elaborated by the substitution of the liquid inside a gel without any significant change in their structure. They also exhibit other advantages, including low density, tunable chemical properties, thermal resistance, and high loading capacity, which makes them appropriate for a wide range of applications, including water treatment, thermal insulation, the food industry, and medical and pharmaceutical applications, among others [17,18,19]. Consequently, aerogels are attracting increasing interest as a means of delivering a range of active compounds for therapeutic drugs, among other applications [20]. There are several methods that can be employed for the fabrication of these scaffolds, such as 3D bioprinting, electrospinning, or the use of hydrogel [21,22,23,24]. The latter method, commonly referred to as phase separation, has gained significant traction due to its capacity to absorb substantial quantities of water. It typically necessitates a freeze-drying process to sublimate the solvent, ultimately leading to the formation of porous structures [23].

Specifically, the formation of aerogels involves the use of a variety of raw materials, such as allotropic carbon, metal oxides, or polymers, which can be synthetic or natural. Nevertheless, the use of natural polymers, also designated as biopolymers, which are renewable and plentiful, is currently experiencing a surge in interest due to their favorable properties, including exemplary cell attachment, biodegradability, and biocompatibility [2,18]. Biopolymers can be classified into two main categories: polysaccharides and proteins. Polysaccharides include substances such as alginate, starch, cellulose, chitin, chitosan, and agar. Proteins, on the other hand, encompass substances like gelatin, collagen, and soy [2,25,26]. These polymers facilitate the formation of both covalent and non-covalent chemical bonds, thereby overcoming the limitations of other materials in terms of cell adhesion and cellular attachment [7].

Collagen (CG) is a protein that plays a pivotal role in the maintenance of the ECM. It is one of the most abundant proteins in this matrix, comprising three polypeptide chains that are linked together to form a triple-helix structure [27,28]. In particular, there are up to 29 types of collagens, although the most abundant is type I collagen [3]. This is found in bones, teeth, skin, and ligaments. This fibrous protein is constituted by proline, glycine, and hydroxyproline [3,28] and has been widely used in recent years for the development of novel scaffolds which combine excellent biocompatibility, low toxicity, and osteogenic induction properties [29,30,31,32,33]. Considering previous studies, collagen is a biopolymer that has been used for the formation of aerogel that supports bone regeneration as a result of its ability for bone biomineralization activity as well as high rate of cell proliferation [34] or for cartilage tissue repair due to its geometry, which is similar to the one that is possessed by the biopolymer, among others [35,36].

On the other hand, chitosan (CH) is a natural polymer derived from the deacetylation and enzymatic degradation of chitin, which allows for the utilization of food waste (especially shells from crustaceans and insects). This polysaccharide is notable for the presence of amino groups in its chemical structure and for its abundance and environmentally friendly nature [37,38,39]. Moreover, the utilization of this polysaccharide has been demonstrated to promote additional properties, such as antiviral, antibacterial, or antifungal characteristics, which are essential for tissue engineering [40]. This has been evidenced by previous studies [41,42,43]. For instance, the incorporation of chitosan allowed the creation of different type of scaffolds, such as temperature-sensitive systems, whose application can be found in several areas, such as neurodegenerative diseases, joint diseases, and dentistry, among others [44,45,46].

In any case, the combination of these biopolymers (CG and CH) presents several disadvantages, including poor mechanical properties [29,32,40]. Consequently, researchers are attempting to develop novel alternatives, such as polymer blending [40,47] or the incorporation of nanoparticles (NPs) [36,48], with the objective of attaining the desired properties. In this context, nanomaterials are refined as a wide range of materials whose dimensions are within the range of 1–100 nm. They are obtained from the manipulation of materials through chemical or physical processes [49,50]. The properties of this type of material are dependent on its size due to the large number of superficial atoms in comparison to its volume, which results in a more active surface [51,52]. The synthesis of these materials can be achieved through a variety of methods, depending on the desired properties and intended applications. These methods can be broadly classified as either top-down (e.g., mechanical grinding and laser ablation) [53,54] or bottom-up techniques (e.g., electric arc, flame synthesis, vapor phase deposition, microemulsion, sol–gel, colloidal synthesis, and biological synthesis) [55,56,57,58,59,60]. Thus, Rahman et al. developed aerogels reinforced with iron oxide NPs through freeze-drying, resulting in materials with a high specific surface area and stability in water [61]. Nevertheless, the integration of these NPs is particularly promising in the biomedical field, where they can facilitate the controlled release of drugs, enhancing their therapeutic efficacy and biological, mechanical, and electrical properties [62]. However, among all the nanoparticles, magnetite is one of the most frequently used ferrites due to its nontoxicity, allowing the formation of drug delivery systems as a result of the increase in the affinity and targetability for cells [63]. Specifically, the addition of magnetite into gel matrix promotes the formation of a highly porous gel [64], as recorded in previous studies where this compound was added into a sol–gel solution, improving blood absorption and mild cytotoxic effects [36], for the development of scaffolds for cardiac tissue engineering [65] or for the formation of hybrid biopolymer-based aerogels, which promotes the adsorption of heavy metal ions and forms a high porous interconnected matrix [66].

Therefore, the objective of this work was the development of a preliminary study for the fabrication of different aerogels modified with nanoparticles. Thus, hybrid aerogels based on collagen and chitosan were created using the phase separation method, reinforcing them with iron oxide nanoparticles obtained through two different methods (chemical or green synthesis). The physicochemical, mechanical, and functional properties of these aerogels were evaluated in order to compare the different systems. Thus, the main novelty of this article lies in the combination of nanoparticles for the formation of collagen–chitosan-based hybrid biomaterials and the comparison of the properties observed in the modified materials with different iron oxide nanoparticles.

## 2. Materials and Methods

### 2.1. Materials

The biopolymers used for the preparation of the aerogels were type I collagen from pigs (HI95), with a protein content higher than 95 wt%, supplied by Essentia Protein Solutions S.A. (Gråsten, Denmark) and low-molecular-weight chitosan (molecular weight (MW) of 130,000 g/mol and with a deacetylation degree of 75–85%), supplied by Sigma Aldrich (San Luis, MO, USA). The collagen was selected due to its high protein content, while the chitosan was chosen for its ability to form stable gels at low concentrations. Furthermore, 0.05 M acetic acid at pH 3.2 was used as a solvent to facilitate the solubilization of the biopolymers.

Additionally, chemically synthesized (Ch-NPs) and environmentally friendly synthetized (Gr-NPs) magnetic iron oxide nanoparticles (mixture of Fe_2_O_3_ and Fe_3_O_4_) were included in this study, sourced from previously published works [67,68]. The Gr-NPs contained 94.6% Fe_3_O_4_ and 3.4% Fe_2_O_3_, with an average diameter of 6.6 ± 4.9 nm. The Ch-NPs were composed of 86.5% Fe_3_O_4_ and 13.3% Fe_2_O_3_, with an average diameter of 16.8 ± 1.4 nm. These nanoparticles exhibit magnetic properties and stability that make them suitable for the enhancement of the structural and functional properties of biopolymeric aerogels.

### 2.2. Aerogel Processing

**Figure 1:**
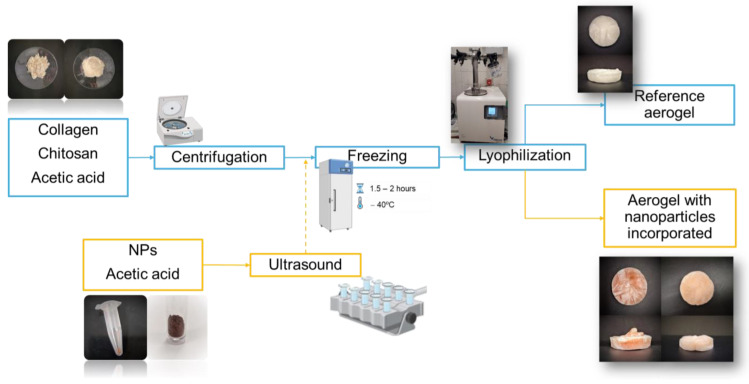
Synthesis of collagen–chitosan-based aerogel (reference system and systems with nanoparticles incorporated).

The aerogels were obtained through a processing method based on the methodology proposed by Perez-Puyana et al. [69], as shown in Figure 1. Thus, an aerogel without nanoparticles (AG-Ref) was produced, comprising a total biopolymer content of 2 *w*/*v*% (1 *w*/*v*% of CG and 1 *w*/*v*% of CH). The biopolymers, together with the 20 mL of solution, were placed in a Sigma 3–18k centrifuge (MEDIFRIGER BL-S, J.P Selecta, Barcelona, Spain) and subjected to centrifugation at 10,000 rpm and 4 °C for a period of 7 min. Subsequently, the samples were frozen at −40 °C for 1.5–2 h and subsequently placed in a freeze-dryer (LyoQuest, TELSTAR, Barcelona, Spain) for 24 h at 0.01 mbar and −80 °C to sublimate the solvent.

The ratio of the base mixture remained unchanged for the aerogels with incorporated nanoparticles. Therefore, the same quantities of collagen and chitosan were used. The main difference in this case is that only 15 mL of 0.05 M acetic acid together with the weighed biopolymers were centrifuged. The remaining 5 mL was combined with 8 mg of nanoparticles (2 *w*/*v*% with respect the total biopolymer content) in a test tube. This test tube was placed in an ultrasound machine (J.P. Selecta S.A., Spain) for 15 min to facilitate the dispersion of the nanoparticles in the solvent. Both samples (biopolymer solution after centrifugation and nanoparticle suspension) were mixed before the freezing step.

### 2.3. Aerogel Characterization

#### 2.3.1. Mechanical Properties

Dynamic compression tests were conducted for the purpose of characterizing the mechanical properties of the aerogels. For this purpose, an RSA3 rheometer (TA Instruments, New Castle, DE, USA) with a plate–plate geometry (dia. of 15 mm) was used. Firstly, strain sweep tests were carried out at 1 Hz within a strain range of 2.5·10^−4^% to 2.5% to determine the linear viscoelastic range and the critical strain (last strain in the linear viscoelastic range) of the aerogels. Subsequently, frequency sweep tests were performed between 0.02 and 20 Hz at a constant strain within the linear viscoelastic range, with the objective of obtaining the values of the elastic modulus (E′), the viscous modulus (E″), and the loss tangent (tan δ = E″/E′) as a function of frequency.

#### 2.3.2. Optical Properties: Color Measurements

A colorimetry test was performed to carry out a color analysis of the samples fabricated, using a high-precision colorimeter MERHOVO (model NR110). Thus, the values of L* (i.e., lightness) and a* and b* (red, green, blue, and yellow in CIELAB color space) were found for each sample as the average of five scans. In particular, b* drifts from negative values (blue) to positive values (yellow), whereas a* drifts from negative values to positive values associated with green and red, respectively.

#### 2.3.3. Scanning Electron Microscopy (SEM)

To analyze the microstructure of the aerogels, a scanning electron microscope, Zeiss EVO (Zeiss, Oberkochen, Germany), with a secondary electron detector was used with an accelerating voltage of 10 kV. The samples were previously coated with a layer (less than 10 nm thick) of Pd/Au and treated with 1% osmium vapor for 8 h to fix the scaffold structure and improve the quality of the obtained micrographs. These images were analyzed using ImageJ software, version 1.54g (National Institute of Health, Bethesda, MD, USA).

On the other hand, the total porosity of the aerogels was calculated based on the method of indirect measurement of the free volume inside the scaffold [70]. The four types of aerogels were measured and weighed to calculate their densities (ρ_scaffold_). The total porosity was obtained using Equation (1):

(1) $$\varepsilon \left(\%\right)=\left(1-\frac{\rho_{scaffold}}{\rho_{material}}\right)\times 100$$

where ρ_material_ is the average density of chitosan and collagen.

#### 2.3.4. Functional Characterization

To evaluate the antioxidant activity of the aerogels, a previous protocol was followed [67,68], which was originally applied to the nanoparticles. Briefly, 4 mg of each aerogel sample was dissolved in a mixture containing 1 mL of DMSO and 1 mL of DPPH solution. Then, the samples were stirred vigorously for 30 s and subsequently left in darkness for 30 min. Finally, the absorbance was measured at 517 nm in a spectrophotometer U-1100 (Hitachi, Chiyoda, Japan). The inhibition percentage was calculated using Equation (2):

(2) $$Inhibition(\%)=\left(\frac{\mathrm{Absorbance}\mathrm{of}\mathrm{control}-\mathrm{Absorbance}\mathrm{of}\mathrm{sample}}{\mathrm{Absorbance}\mathrm{of}\mathrm{control}}\right)\times 100$$

This equation allows for the quantification of antioxidant activity by comparing the absorbance of the control solution with that of the aerogel samples.

The thermal stability of the aerogels was studied at 50 °C and 100% relative humidity. For this purpose, one-quarter of each scaffold was cut and placed on Petri dishes. They were then placed in a double-bottomed container. The lower part was filled with a solution of salt in water, achieving a humidity of 100%, while the upper part was filled with the four samples. Finally, the container was placed in an oven at 50 °C. Photos were taken at 0, 1, 2, 12, 24, 48, and 60 h to observe the degradation process of the aerogels over time.

### 2.4. Statistical Analysis

At least three replicates of each sample were obtained in order to evaluate the replicability and reproducibility of the results. Significant differences were evaluated with *t*-tests at a confidence level of 95% (*p* < 0.05).

## 3. Results and Discussion

### 3.1. Aerogel Characterization

#### 3.1.1. Macrostructural Appearance of the Aerogels

**Figure 2:**
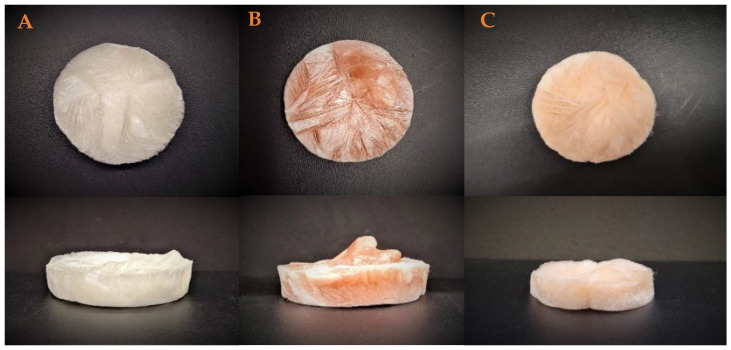
Macrostructural appearance of the aerogels elaborated. (**A**) Reference, (**B**) chemical nanoparticles incorporated, and (**C**) green nanoparticles incorporated.

Figure 2 shows the macrostructural appearance of the elaborate aerogels. As can be seen, the reference system (AG-Ref, Figure 2A) exhibits a white coloration in accordance with its composition. Nevertheless, the incorporation of nanoparticles results in a change in coloration to orange. Thus, the incorporation of chemical nanoparticles (Ch-NPs, Figure 2B) or green nanoparticles (Gr-NPs, Figure 2C) results in the formation of an orange hue in the systems, with the Ch-NPs systems exhibiting a darker orange coloration. This darkening may be attributed to the larger size of the Ch-NPs, which allows for greater color prominence within the structure.

#### 3.1.2. Optical Properties: Color Measurements

As shown previously, there are slight differences between the macrostructural appearance of the different samples. The values associated with the color parameters are shown in Table 1. The reference system associated with a collagen–chitosan-based aerogel presented a whitish color as shown in the values of L*, a*, and b*. Regarding the samples which incorporate nanoparticles, there is a darkening of the samples, as well as an increase in a* and b* values as a result of the addition of brown-orange nanoparticles.

#### 3.1.3. Mechanical Properties

**Figure 3:**
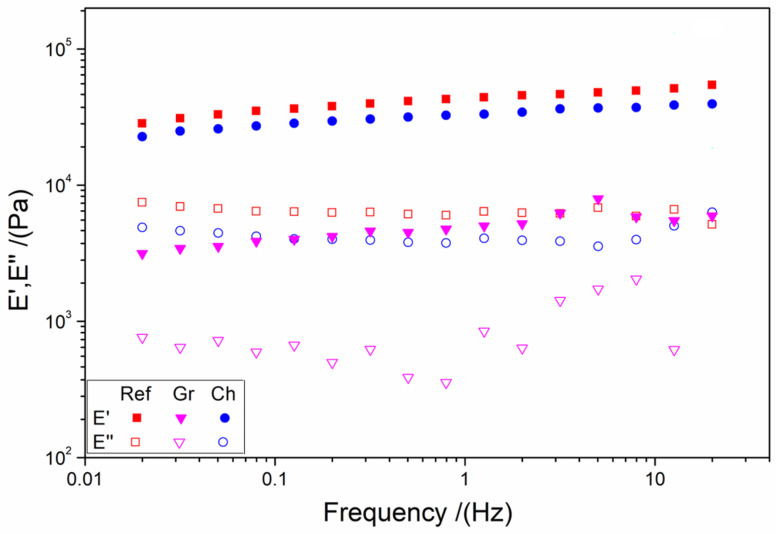
Frequency sweep tests of the different aerogels: reference (Ref), chemical nanoparticles included (Ch), and green nanoparticles included (Gr).

The results of the frequency sweep tests of the different systems are shown in Figure 3. It can be observed that the Ref and Ch systems exhibit modulus values (for both E′ and E″) that are practically stable at the entire frequency range studied. Nevertheless, the Gr system presents a slight variation in these values, particularly in E″. This behavior could be attributed to the inherently unstable nature of the Gr-NPs, which is a consequence of the presence of polyphenols during the manufacturing process. The polyphenols remain on the surface of the nanoparticles, thereby creating a system that is more unstable.

Table 2 shows the values of the elastic modulus at 1 Hz (E′_1_), critical strain (ε_crit_), and loss tangent at 1 Hz (tan δ_1_) in order to facilitate the comparison between the systems. All systems exhibit solid character (tan δ_1_ < 1) without significant differences. On the other hand, the elastic modulus was markedly higher for the Ref and Ch systems, and it was observed that these values are similar to those obtained for applications such as brains (15 kPa), kidneys (50 kPa), pancreas, iris, or lungs (80 kPa) as shown in the literature [13,71]. This different behavior is similarly evident in the critical strain, wherein the Ref system exhibits the lowest value and the Gr system the highest one. These results may be attributed to the presence of polyphenols in the Gr systems, which have been demonstrated to generate ionic forces that could impede the interaction of NPs with the biopolymers.

#### 3.1.4. Scanning Electron Microscopy (SEM)

**Figure 4:**
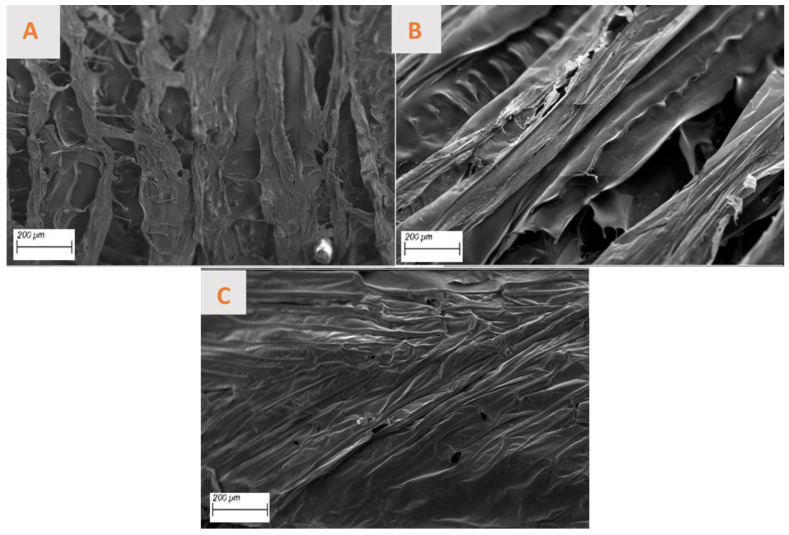
Micrographs of different aerogels made: (**A**) reference, (**B**) chemical nanoparticles included, and (**C**) green nanoparticles incorporated.

The micrographs of the three aerogels are presented in Figure 4. They show an irregular structure, characterized by the presence of micro- and macro-pores. The Ref and Ch aerogels show very similar laminar structures (Figure 4A,B), with a defined directionality. This may account for the minimal difference in modulus observed in the rheological study. The Gr aerogel also exhibits a laminar structure, but not directional, and displays greater heterogeneity (Figure 4C). The low values of the elastic and viscous moduli can be attributed to the lack of optimal stacking, as a result of the existence of a higher concentration of solids in the matrix that alter the morphology of the aerogels, promoting a minimization in the dimension of the pore opening as recorded in previous works [72,73]. In this sense, different works reveal that this irregular shape is typical of this type of material, forming an interconnected porous structure that is necessary for enhancing cell migration and the transportation of biomolecules [74,75,76]. This structure, which is composed of the superposition of different layers, may be linked to the gelation procedure that is needed for the formation of the aerogel by the lyophilization of a hydrogel [77].

Regarding porosity, while the Ref and Ch aerogels display a multitude of pores exhibiting heterogeneous sizes and shapes (with a mean porosity of 46.5 ± 15.8 nm and 29.4 ± 17.4 nm, respectively) and a relatively uniform dispersion. On the other hand, Gr aerogels are devoid of pores, with the existing ones being uniformly minute. This suggests that the aerogels studied possess an adequate pore size, which would facilitate cell regeneration and growth in their potential application as a biomaterial. Consequently, to facilitate a more precise comparison of the porosity of the aerogels, the percentage of total porosity of each material has been calculated and is presented in Table 3. In general, all aerogels show adequate porosity values (>98%), which are greater than the necessary 85% for most of the applications [78] and which can act as a favorable point for applications such as bone and cartilage regeneration as shown in the literature [79]. As can be observed in the table above, the theorical porosity of AG-Gr is the highest, which does not correspond to what has been discussed of the SEM images. This is due to the fact that the calculated porosity refers to the total volume of the aerogel, while the SEM technique allows us to study only the layer of the material and pores that can be found inside the scaffold.

#### 3.1.5. Functional Characterization

Table 4 shows the results obtained for this study regarding the antioxidant ability of the different aerogels synthesized. It can be observed that all the aerogels possess similar values associated with the percentage of the inhibition values, showing how they are capable of resisting oxidation in a way better than the white sample; the aerogels contribute to the reduction in reactive oxygen species, which can be found in the wound and which can promote the proliferation of inflammatory cells. Thus, in this way, it would be possible to overcome the inflammatory phase and achieve faster wound healing. However, if the values displayed in Table 3 are compared, it can be seen that the incorporation of nanoparticles aggravates the antioxidant capacity of the aerogels, gainsaying the results obtained by the nanoparticles which show an enhancement in the inhibition percentage [51,80]. Antioxidant capacity is a complex property which can be possessed by multiple types of groups [81]. It is possible that a compound that has a greater amount of antioxidant groups may not have a good antiradical function. The effectiveness of the compounds depends on multiple factors, such as their structural chemical properties; their temperature; the characteristics of the substance that is oxidized, its concentration, and its location in the system (interfacial distribution); the presence of pro-oxidant compounds; the physical state of the system; and the kinetics of the reaction [82,83].

**Figure 5:**
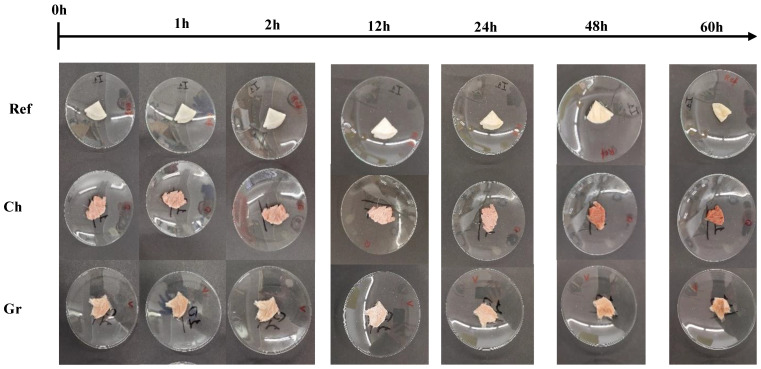
Temporal evolution of thermal stability for the aerogels studied: reference (Ref), chemical nanoparticles included (Ch), and green nanoparticles included (Gr).

In the same way, Figure 5 illustrates the results obtained in the thermal stability study. Therefore, during the initial 12 h period, aerogels maintain their structural integrity, exhibiting no discernible alterations. However, after 24 h, a slight change in color is evident, which is attributed to the biodegradation process of the system due to the presence of high humidity combined with temperatures. Similarly, it can be seen that a notable volume loss occurs within the initial 48 h.

**Figure 6:**
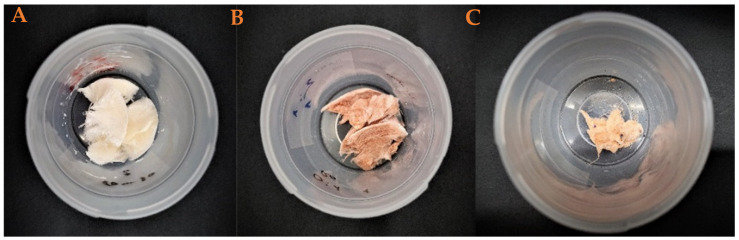
Macroscopic appearance of the pieces of the aerogels after 3 weeks from their fabrication: (**A**) reference, (**B**) chemical nanoparticles included, and (**C**) green nanoparticles included.

Furthermore, a sample of each synthetized aerogel was maintained at room temperature and in laboratory conditions in order to evaluate their stability during storage. Figure 6 shows these systems. As can be seen, the incorporation of green nanoparticles to the aerogels promotes their own degradation after 3 weeks. So, unlike other aerogels that have remained in the same state since their manufacture, the Gr aerogel contracted, thus losing its porosity and other properties. This could be due to the polyphenols present in the system, giving the aerogel some advantages (for example, greater deformability) but also some disadvantages such as instability. This is caused by the interaction of the charges of these polyphenols.

## 4. Conclusions

Collagen–chitosan-based aerogels were successfully developed using phase separation and lyophilization, incorporating nanoparticles produced by different methods. The aerogels exhibited frequency-independent behavior. The addition of nanoparticles reduced biopolymer interactions, and green nanoparticles specifically decreased elasticity due to their tendency to aggregate with polyphenol surface charges. SEM studies revealed amorphous structures.

For tissue engineering applications, the aerogels demonstrated porosity values above 98%, supporting cell proliferation, a key factor for wound healing and for other applications such as in the field of tissue engineering (for cartilage regeneration as aforementioned). However, the incorporation of nanoparticles did not enhance antioxidant capacity (obtaining values of 47.60, 46.17, and 43.96% for reference systems and aerogels that incorporate chemical and green nanoparticles, respectively), likely due to aggregation and magnetic interactions, limiting their dispersion and reducing the effective surface area within the biopolymeric matrix.

To optimize aerogel properties, further research is necessary, including studies on skin adhesion, nanoparticle distribution within the matrix, and sample stability at various pH levels, critical for compatibility with the human body. Complementary tests of antioxidant capacity, such as electrochemical methods, should be conducted. Additionally, their potential as wound dressings require in vitro analyses of cell viability, fixation, proliferation, and differentiation, as well as antibacterial property assessments and experimental wound healing models.

## Figures and Tables

**Table 1:** Color parameters for each system: reference (Ref), chemical nanoparticles included (Ch), and green nanoparticles included (Gr). Different letters or symbols mean significant differences (*p* < 0.05).

Sample	L*	Δ*L*	b*	Δ*b*	a*	Δ*a*
Ref	83.9 ± 6.8 ^a^	-	2.5 ± 0.5 ^A^	-	10.2 ± 2.0 ^α^	-
Ch	70.7 ± 2.4 ^b^	−13.2	18.3 ± 4.0 ^B^	15.8	20.9 ± 6.3 ^β^	10.8
Gr	77.2 ± 6.6 ^b^	−6.7	12.2 ± 1.0 ^C^	9.7	23.1 ± 1.0 ^β^	13.0

**Table 2:** Elastic modulus and loss tangent at 1 Hz (E′_1_ and tan δ_1_, respectively) and critical strain (ε_crit_) values for each system: reference (Ref), chemical nanoparticles included (Ch), and green nanoparticles included (Gr). Different letters or symbols mean significant differences (*p* < 0.05).

Sample	E′_1_ (kPa)	tan δ_1_ (-)	ε_crit_ (%)
Ref	27.46 ± 18.24 ^α^	0.10 ± 0.05 ^a^	0.34 ± 0.15 ^A^
Ch	25.74 ± 10.19 ^α^	0.13 ± 0.01 ^a^	0.79 ± 0.01 ^B^
Gr	5.79 ± 4.43 ^α^	0.15 ± 0.02 ^a^	1.63 ± 0.53 ^C^

**Table 3:** Theoretical porosity values of each aerogel studied: reference (Ref), chemical nanoparticles included (Ch), and green nanoparticles included (Gr). Different letters mean significant differences (*p* < 0.05).

Sample	Porosity (%)
Ref	98.3 ± 0.1 ^c^
Ch	98.6 ± 0.1 ^b^
Gr	99.5 ± 0.1 ^a^

**Table 4:** Percent inhibition values for each system made: reference (Ref), chemical nanoparticles included (Ch), and green nanoparticles included (Gr). Different letters mean significant differences (*p* < 0.05).

Sample	Inhibition (%)
Ref	47.60 ± 0.04 ^a^
Ch	46.17 ± 0.61 ^b^
Gr	43.96 ± 0.03 ^c^

## Data Availability

All data and materials are available on request from the corresponding author. The data are not publicly available due to ongoing research using a part of the data.
